# Fear of hypoglycaemia: defining a minimum clinically important difference in patients with type 2 diabetes

**DOI:** 10.1186/1477-7525-7-91

**Published:** 2009-10-22

**Authors:** Tom Stargardt, Linda Gonder-Frederick, Karl J Krobot, Charles M Alexander

**Affiliations:** 1 Health Services Management, Munich School of Management, Munich University, Germany; 2 Institute of Health Economics and Health Care Management, Helmholtz Zentrum Munich, Germany; 3 Department of Psychiatry and Neurobehavioral Sciences, University of Virginia, Charlottesville, VA, USA; 4 Outcomes Research, MSD Sharp & Dohme GMBH, Haar, Germany; 5 Outcomes Research, Merck & Co, Inc, Whitehouse Station, NJ, USA

## Abstract

**Background:**

To explore the concept of the Minimum Clinically Important Difference (MID) of the Worry Scale of the Hypoglycaemia Fear Survey (HFS-II) and to quantify the clinical importance of different types of patient-reported hypoglycaemia.

**Methods:**

An observational study was conducted in Germany with 392 patients with type 2 diabetes mellitus treated with combinations of oral anti-hyperglycaemic agents. Patients completed the HFS-II, the Treatment Satisfaction Questionnaire for Medication (TSQM), and reported on severity of hypoglycaemia. Distribution- and anchor-based methods were used to determine MID. In turn, MID was used to determine if hypoglycaemia with or without need for assistance was clinically meaningful compared to having had no hypoglycaemia.

**Results:**

112 patients (28.6%) reported hypoglycaemic episodes, with 15 patients (3.8%) reporting episodes that required assistance from others. Distribution- and anchor-based methods resulted in MID between 2.0 and 5.8 and 3.6 and 3.9 for the HFS-II, respectively. Patients who reported hypoglycaemia with (21.6) and without (12.1) need for assistance scored higher on the HFS-II (range 0 to 72) than patients who did not report hypoglycaemia (6.0).

**Conclusion:**

We provide MID for HFS-II. Our findings indicate that the differences between having reported no hypoglycaemia, hypoglycaemia without need for assistance, and hypoglycaemia with need for assistance appear to be clinically important in patients with type 2 diabetes mellitus treated with oral anti-hyperglycaemic agents.

## Background

The goal of treatment of diabetes is to achieve glycemic control in order to prevent long-term micro- and macro-vascular complications. Due to the progressive nature of beta cell failure in spite of treatment with anti-hyperglycemic agents, HBA_1c _levels slowly rise even after an initial fall in patients with type 2 diabetes [1-3]. Therefore an increasing number of patients eventually need to be treated with combination medication regimens and/or insulin [4,5].

One of the major challenges in the treatment of diabetes is to achieve glycemic control while avoiding episodes of hypoglycaemia [6-8]. Hypoglycaemia is due to excess insulin levels in relation to circulating glucose. Etiologies may include missed meals, physical activity, drug interactions, or the anti-hyperglycaemic medication regimen [6,7,9-12]. If untreated, hypoglycaemia may affect brain function, both cognitive and motor. With severe hypoglycaemia, convulsions, coma or death may occur [13,14]. Recurrent severe episodes of hypoglycaemia can lead to behavioral changes [6], cognitive impairment [15], and unawareness of hypoglycaemia [16]. Because of these negative consequences, patients may develop psychological fear of hypoglycaemia. This fear can become phobic [17], reduce quality of life [18], and impact adherence with diabetes management [7,12,13]. Fear of hypoglycaemia decreases after interventions such as behavioral programs and islet cell transplant surgery [19-21].

However, little is known about fear of hypoglycaemia in patients with type 2 diabetes treated with combinations of oral agents. This study therefore explores the concept of the Minimum Clinically Important Difference (MID) for the Worry Scale of the Hypoglycaemia Fear Survey (HFS-II) in a large sample of patients with type 2 diabetes treated with combinations of metformin and a sulphonylurea or metformin and a glitazone. According to the definition of Jaeschke, et al., MID is "the smallest difference in score [in the domain of interest] which patients perceive as beneficial and which would mandate [in the absence of troublesome side-effects and excessive cost] a change in the patient's management" [22]. As even very small absolute differences in patient-related outcomes such as HFS-II can become statistically significant given large group sizes, it is very important to find a threshold that indicates whether a difference in score is clinically meaningful or not [23].

This is the first study to develop a MID for fear of hypoglycaemia. The MID was then used to quantify and evaluate difference in HFS-II scores between patients who reported no hypoglycaemic episodes, hypoglycaemic episodes without need for assistance, and hypoglycaemic episodes with need for assistance.

## Methods

### Setting and design

An observational multi-centre study was conducted in Germany. Patients were recruited in a convenience sample of 92 sites by their physicians, either GPs or diabetologists. Data were collected between October and December 2005. To be included, patients were required to be diagnosed with type 2 diabetes, 35 years or older, and had to be treated during the 6 months prior to the study with either a combination of metformin and a glitazone, or with a combination of metformin and a sulphonylurea. Patients were not eligible if they had been treated with insulin in the past, were taking part in a clinical trial, or were being treated for HIV or hepatitis. Data were collected using medical records review and questionnaires. Physicians were asked to provide information on the participant's medical history, baseline laboratory measures, and diabetes medications used during the six months prior to the study. After informed consent, participants completed the Treatment Satisfaction Questionnaire for Medication version 1.4 (TSQM) [24], a socio-demographic questionnaire, and the Worry Scale of the HFS-II. In addition, patients were asked about severity of hypoglycaemic episodes during the previous six months.

### Hypoglycaemia Fear Survey-II

The HFS-II is a 33-item questionnaire with two subscales that measure 1) behaviours to avoid hypoglycaemia and its negative consequences and 2) worries about hypoglycaemia and its negative consequences. Responses are made on a 5-point Likert scale where 0 = Never and 4 = Always. This study used the 18-item Worry subscale which has a score range of 0 - 72 with higher scores indicating increased fear of hypoglycaemia. The HFS-II is a widely used measure in clinical trials, has been translated into more than 20 languages, and has demonstrated reliability and validity [25,26].

### Construction of MID

In general, studies have recommended using a variety of methods to determine MID [27,28]. In this study, we used a variety of established distribution-based and anchor-based methods. With regard to distribution-based methods, we used the score's standard error of measurement, the score's standard deviation multiplied by the square root of 1 minus Cronbach's alpha [29,30], and multiples of the score's standard deviation: 0.5, 0.20, 0.30, and 0.33 [23,27-29,31] to calculate MID. In addition, MID was computed as 8% of the theoretical score ranges (HFS-II: 72 points) [28].

As one of the patient-based anchors, we used treatment satisfaction to determine MID - due to its proximity to self-reported treatment success. Our MID is based on the assumption that patients who are not satisfied with their treatment are more likely to either not adhere to medications, or to complain to their doctors, which in turn may lead to a change in medications. We assume that treatment satisfaction is closely related to self-reported treatment success (i.e. 'worsening of condition' vs. 'no change in condition' or 'small improvement' vs. 'no change'), a concept that has been recommended to determine MID in many other studies [27,28,32]. Treatment satisfaction was measured as the response to the seven-point scaled TSQM question 14 'Taking all things into account, how satisfied or dissatisfied are you with this medication'. The answer categories that were less than 'satisfied' (e.g. 'somewhat satisfied', 'dissatisfied', 'very dissatisfied', and 'extremely dissatisfied') were combined as 'less than satisfied'. We thus determined MID for the Worry Scale of HFS-II by taking the difference in score means between patients who were 'satisfied' and patients who were 'less than satisfied' with their medication. As negative side effects, especially increased hypoglycaemia, may be also important and can lead to decreased medication adherence or a change in regimen, we also used responses to TSQM questions 6, 'To what extent do the side effects interfere with your physical health and ability to function', and TSQM question 8, 'To what degree have medication side effects affected your satisfaction with the medication', as anchors to determine MID. However, questions 6 and 8 of the TSQM were only answered by the smaller subsample of patients that experienced side-effects. Difference in means of the Worry Scale of HFS-II of patients who stated that they were 'somewhat' affected and patients that stated that they were 'quite a bit' or 'a great deal' affected were compared.

### Severity and fear of hypoglycaemia

According to the recommendations of the American Diabetes Association [16], severity of hypoglycaemic episodes were categorized as 1) mild (little or no interruption of activities; no treatment assistance needed), 2) moderate (some interruption of activities; no assistance needed) and 3) severe (assistance of a third party needed). A fourth category, very severe hypoglycaemia, was added to capture episodes that required medical assistance. In the questionnaire, the different levels of severity of hypoglycaemia were defined for the patients who were asked if they experienced any of the four severity categories of hypoglycaemia during the last 6 month. If a patient reported episodes of hypoglycaemia at different levels of severity, the patient was classified according to the most severe episode reported. Following recommendations by other studies [11,12,16,33], the categories mild and moderate were aggregated to 'hypoglycaemia without need for assistance' and the categories severe and very severe were aggregated to 'hypoglycaemia with need for assistance'. Results for MID were then applied to evaluate whether the difference between not having hypoglycaemic episodes and having different levels of severity of hypoglycaemic episodes is clinically important in this study population. In addition the mean scores of the Worry Scale of HFS-II for patients who reported hypoglycaemic episodes during the last six month were compared to patients who did not report hypoglycaemic episodes. One-way analysis of variance and Tukey's honestly significant differences test were used to test for statistical significance of differences in mean scores. A p-value of 0.05 was considered statistically significant. We also calculated Cohen's d statistics as a marker of effect size for differences in HFS-II. Statistical analyses were conducted using SAS version 9.1.3.

## Results

From 402 patients recruited at 92 sites, two were excluded because they did not meet inclusion or exclusion criteria, and eight were excluded because they had not fully completed the Worry Scale of HFS-II. The final study population thus comprised 392 patients, of whom 268 patients were treated with metformin and a sulphonylurea, and 107 patients with metformin and a glitazone. For 17 patients it was unknown which of the two medication regimens they were on. As the differences between patients treated with metformin and a sulphonylurea and patients treated with metformin and a glitazone did not reach statistical significance for hypoglycaemia (p = 0.1127) or HFS-II scores (p = 0.5222), the medication groups were combined for subsequent analysis.

### Patient characteristics

Mean age (SD) of the study population was 62.7 (10.6) years. 42.6% were female. 28.9% of patients had a history of macrovascular complications, while 16.4% had a history of microvascular complications. Further details are given in table 1. Average score (SD) of the Worry Scale of HFS-II for the entire sample was 8.06 (10.4), with a minimum of 0 and a maximum of 51. While 125 patients were extremely satisfied with their medication (TSQM question on treatment satisfaction), 113 patients, 100 patients, and 44 patients reported being 'very satisfied', 'satisfied', and 'somewhat satisfied' with their medication, respectively. The number of patients who were 'dissatisfied', 'very dissatisfied', and 'extremely dissatisfied' were only 4, 5, and 1, respectively.

**Table 1:** Patient characteristics

	**Mean (SD)**
Age, years	62.7 (10.6)
Gender	
Female	42.6%
Male	57.4%
Married	62.8%
HBA_1c_	7.24 (1.23)
HBA_1c _at goal [6.5%]	29.1%
Weight (kg)	87.3 (15.8)
BMI (kg/m^2^)	29.6 (4.6)
History of complications	
Macrovascular	28.9%
Microvascular	16.4%
Duration of diabetes, years	
<4	15.1%
4-6	24.6%
7-9	31.7%
> 9	28.6%

### Construction of MID

MIDs determined according to distribution-based methods varied widely (see table 2). While the MID for the Worry Scale of HFS-II based on the standard error of measurement was 2.0, MID based on 8% of the theoretical score range was 5.8. MIDs based on 0.2, 0.30, 0.33 and 0.5 multiplied by standard deviation, respectively, varied between 2.1 and 5.2 for the Worry Scale of HFS-II.

**Table 2:** Distribution- and anchor-based MIDs for the Worry Scale of HFS-II.

**Method**	**MID for HFS-II**
*Distribution-based MIDs*	
Standard deviation multiplied by 0.2	2.1
Standard deviation multiplied by 0.3	3.1
Standard deviation multiplied by 0.33	3.4
Standard deviation multiplied by 0.5	5.2
8% of theoretical score range	5.8
Standard error of measurement	2.0
Anchor-based MIDs	
Satisfaction with medication (TSQM question 14)	3.6
Impact of side effects on physical health (TSQM question 6)	3.6
Impact of side effects on satisfaction with medication (TSQM question 8)	3.9

**Figure 1:**
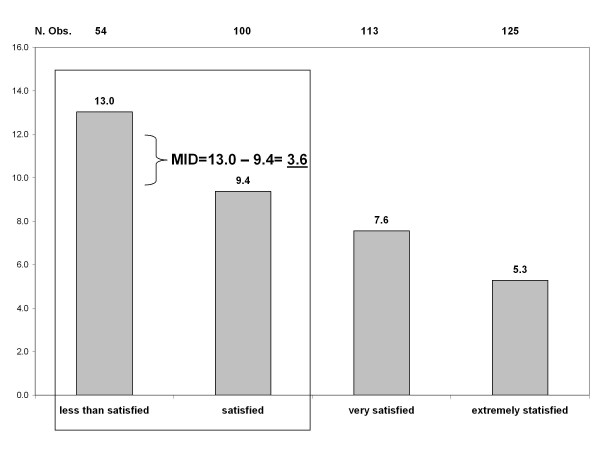
**Mean score of the Worry Scale of HFS-II, by TSQM question 14 on treatment satisfaction, and definition of an anchor-based MID**.

Mean score (+/- SD, n = number of patients in category) of the Worry Scale of HFS-II by satisfaction with treatment was 5.3 (+/- 7.0, n = 125) for patients who were extremely satisfied, 7.6 (+/- 9.4, n = 113) for patients who were very satisfied, 9.4 (+/- 11.7, n = 100) for patients who were satisfied, 13.5 (+/- 14.4, n = 44) for patients who were somewhat satisfied, 16.5 (+/- 13.0, n = 4) for patients who were dissatisfied, 8.4 (+/- 12.8, n = 5) for patients who were very dissatisfied, and 2.0 for a single patient who was extremely dissatisfied. Comparing mean scores of the Worry Scale of HFS-II for patients who were less than satisfied with patients who were satisfied resulted in an MID of 3.6 (see figure 1).

Using the TSQM questions on side effects (questions 6 and 8) resulted in a MID for the HFS-II Worry Scale of 3.6 and 3.9, respectively. The MIDs are based on the answers of 29 patients who were 'somewhat' affected vs. 12 patients who were 'a great deal' or 'quite a bit' affected for TSQM question 6 and on 15 vs. 31 patients for TSQM question 8.

### Severity and fear of hypoglycaemia

**Figure 2:**
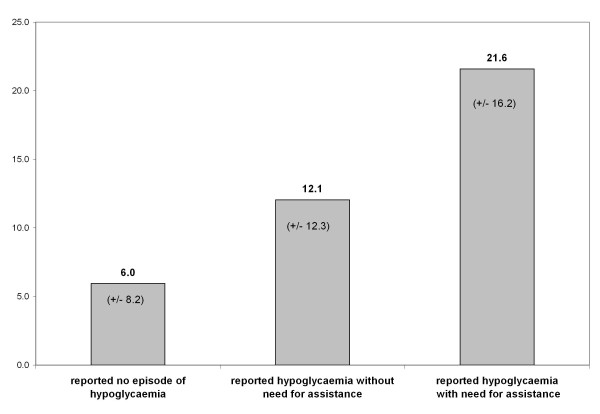
**Mean score (+/- SD) of the Worry Scale of HFS-II, by severity of hypoglycaemia**.

112 patients (28.6% of total sample) reported episodes of hypoglycaemia during the previous 6 months. While 51 and 46 patients reported mild and moderate episodes of hypoglycaemia, 9 and 6 patients reported severe and very severe episodes of hypoglycaemia, respectively. Thus among those reporting hypoglycaemia, 86.6% reported hypoglycaemia without the need for treatment assistance, while 13.4% reported hypoglycaemia with need for assistance. The mean Worry Scale of HFS-II was 6.0 (SD 8.2, 95% CI [5.0; 6.9]) for patients who did not report hypoglycaemic episodes during the previous 6 months, and 13.3 (SD 13.4, 95% CI [10.8; 15.8]) for patients who reported hypoglycaemia during the previous 6 months. The crude difference in the Worry Scale of HFS-II between patients who reported no hypoglycaemia and hypoglycaemia without need for treatment assistance was 6.1 (p = 0.0001, Cohen's d = -0.65 [SD 0.12]). HFS-II scores were also higher in patients who reported hypoglycaemia with need for assistance compared to those who reported hypoglycaemia without need for assistance (p = 0.0100, Cohen's d = -0.74 [SD 0.28]). The size of these effects was generally larger than the MIDs determined by anchor- or distribution-based methods (see figure 2).

## Discussion

HFS-II scores in this study and for this patient group reflected the expected patterns in fear of hypoglycaemia. Patients who reported hypoglycaemia showed more fear than those who did not, and patients who reported more severe episodes of hypoglycaemia showed more fear that those who reported less severe episodes. Based on the MID results of this study, the difference in the Worry Scale of the HFS-II between not having and having reported hypoglycaemia without the need for assistance appears to be clinically meaningful to patients. This is particularly relevant because the vast majority of episodes of hypoglycaemia reported in this study were those which patients managed themselves without the need for assistance from others.

To determine MID, two classes of methods have been discussed in the literature, distribution-based methods and anchor-based methods [34]. Distribution-based methods are based on mathematical calculations that involve standard deviation, score range or Cronbach's alpha. Anchor-based methods are based on judgment of treatment success [35]. Among the group of anchor-based methods, either physician- or patient-based anchors can be applied. While distribution-based methods that involve calculations with the standard deviation, also depend on the heterogeneity of the study population [23], anchor-based methods critically depend on the validity of patients' rating [23] and on choosing response categories that reflect the importance of a change. No gold standard for determining MID currently exists. Therefore we followed the suggestion of Guyatt et al. 2002 that is more accurately to report a range of MID retrieved by different approaches than to recommend a single MID.

Wells et al., had patients compare their own health status with that of their peers. MID was calculated from the difference in score between patients who felt 'somewhat better than other patients' and patients who felt 'about the same' [32]. Another approach is to have patients rate their own improvement due to treatment. Depending on the disease, MID is then calculated as the difference in mean scores between patients who report a 'small improvement' and those who felt 'a little worse', or between patients who report a 'small improvement' and those who reported 'no change' [22,23,29,36]. Walters and Brazier surveyed change in condition over time to establish a MID for quality of life measures, calculated as the changes in score means between patients reporting 'an improvement' and those reporting 'no change' in condition [31]. In our study, one of the anchors chosen to determine MID was based on treatment satisfaction ('less than satisfied' vs. 'satisfied') which we considered closely related to the construct of self-reported treatment success ('no change' vs. 'small improvement') frequently recommended for studies that examine treatment effects [27,30].

The results for the MID using TSQM question 14 on treatment satisfaction (3.6) and TSQM questions 6 and 8 on side effects (3.6 and 3.9) were relatively consistent. Anchor-based MID estimates of the HFS-II were well within the range obtained from distribution-based methods. However, compared to the MID based on treatment satisfaction (n = 154), the MID's based on side effects were derived from the smaller number of patients (n = 29) who reported side effects. Also, hypoglycaemia does not appear to have been the only source for variation in treatment satisfaction. Hypoglycaemia may not have been the only side effect experienced by patients. For these reasons, these results should be considered exploratory. The unexpected low HFS scores for patients who were 'very dissatisfied' (5 patients, HFS-II 12.8) and 'extremely dissatisfied' (1 patient, HFS-II 2.0) compared to patients who were 'dissatisfied' (4 patients, HFS-II 16.5) or 'somewhat satisfied' (44 patients, HFS-II 13.5) may be due in part to the low number of patients in the categories. Another explanation is that perhaps these patients did not adhere to their prescribed medication regimen due to their dissatisfaction and, therefore, had less risk of hypoglycaemia.

While the response categories 'extremely dissatisfied', 'very dissatisfied', 'dissatisfied', and 'somewhat satisfied' were aggregated to 'less than satisfied', we did not aggregate the other response categories of the TSQM question on treatment satisfaction. In our opinion, aggregating 'satisfied', 'very satisfied', and 'extremely satisfied' and comparing these patients to patients 'less than satisfied' would have no longer constituted a *minimum *clinically important difference [27].

It is important to note, however, that our MID estimates may not apply to other types of patients with diabetes [37]. Patients in this study had type 2 diabetes managed by combined oral anti-hyperglycemic medications, and the majority of them (71%) reported no hypoglycaemic episodes in the previous 6 months. Less than 4% reported severe or very severe episodes. Different MID estimates would likely be generated in the subgroup of patients with type 1 diabetes who experience frequent, recurrent episodes of severe hypoglycaemia. Nonetheless, the results of this study suggest that fear of hypoglycaemia, as measured by the HFS-II, can be a useful outcome variable in diabetes health services research, and that even relatively small differences in scores can be clinically meaningful to patients with type 2 diabetes mellitus using oral anti-hyperglycemic medications.

Observational studies can provide valuable information on effectiveness due to real-world settings and larger study populations [38,39]. However, self-reported outcomes from a large number of sites also introduce bias and limitations. The participating physicians may not have always had complete knowledge about parallel prescriptions to their patients and patient's visits to other physicians or hospitals. Eventually, this might have led to incomplete data on patient's medical history, or inclusion of patients who would have otherwise been excluded. Episodes of hypoglycaemia were most likely underreported in our study population, since many patients with diabetes may not always recall or recognize symptoms of hypoglycaemia [6,10,17], or may have limited knowledge about hypoglycaemia itself [40].

## Conclusion

The methodological approach suggested in this study might also be applicable to other patient reported outcomes, in particular, when the MID cannot be based on treatment success. By using the concept of MID, it could be shown that the difference between having reported no hypoglycaemia and having reported hypoglycaemia without need for assistance is clinically meaningful to patients with type 2 diabetes mellitus on oral anti-hyperglycaemic agents. Whether the MID estimates for HFS scores found in this study are applicable to different types of diabetes patients, countries, and cultures should be subject of future research.

## Conflict of interests statement

TS was on a research fellowship sponsored by Merck & Co., Inc, by the time the article was written. LGF has been working under a consultancy agreement for Merck & Co., Inc. LGF has also worked under consultancy agreements or received research grants from Abbott Diabetes Care, Abbott Labs. KJK and CA are employees of Merck & Co., Inc.

## Authors' contributions

KJK conceived the idea to write this paper. TS analyzed the data and drafted the first version of the manuscript. All authors contributed to the conception and design of the study, to interpreting the data, and to writing the manuscript. All authors read and approved the final manuscript.
